# Bond strength in class V cavities: an *in vitro* study on beveling, universal adhesive strategy, and composite type

**DOI:** 10.1590/1678-7765-2025-0608

**Published:** 2026-02-12

**Authors:** Felipe José Ribeiro de Melo, Viviane Rocha, Vivian Leite Martins, Marcos Vinícius Salvador, Adriano Fonseca Lima, Andrea Nóbrega Cavalcanti

**Affiliations:** 1 Escola Bahiana de Medicina e Saúde Pública Curso de Odontologia Salvador BA Brasil Escola Bahiana de Medicina e Saúde Pública, Curso de Odontologia, Salvador, BA, Brasil; 2 Núcleo Innovare Curso de Especialização em Dentística Salvador BA Brasil Núcleo Innovare, Curso de Especialização em Dentística, Salvador, BA, Brasil; 3 Universidade Paulista (UNIP) Pós-Graduação em Odontologia São Paulo SP Brasil Universidade Paulista (UNIP), Pós-Graduação em Odontologia, São Paulo, SP, Brasil

**Keywords:** Dentin-bonding agents, Cavity preparation, Composite resins

## Abstract

Cervical Class V lesions present restorative challenges due to their multifactorial etiology and complex bonding substrates. Objectives: This study evaluated the influence of marginal bevel, bonding using a universal adhesive (etch-and-rinse or self-etch), and the resin composite type (conventional or bulk-fill) on the bond strength of simulated Class V cavities. Methodology: A total of 80 dental fragments (7mm×7 mm×2 mm) were obtained from bovine incisors. Cylindrical cavities with enamel margins and internal dentin walls were prepared centrally in each specimen. Samples were randomly assigned to groups based on three factors: presence or absence of bevel, bonding strategy (etch-and-rinse or self-etch), and resin composite type (conventional or bulk-fill). Groups with bevel received a 1 mm enamel bevel along the preparation margins using a conical diamond bur. Adhesive and restorative procedures were performed following the manufacturer’s recommendations. Bond strength was assessed using a push-out test on a universal testing machine. Data were analyzed using three-way ANOVA and Tukey’s post-hoc test (α=5%). Results: Presence of a marginal bevel did not significantly influence bond strength. The etch-and-rinse bonding strategy resulted in significantly higher bond strength than the self-etch approach, and bulk-fill composites presented superior performance compared with conventional composites. Conclusions: Combining a universal adhesive applied with the etch-and-rinse strategy and a bulk-fill composite proved to be an effective approach for enhancing bond strength with enamel and dentin substrates. Beveling of enamel margins did not improve the bond strength of Class V restorations, suggesting that this procedure may be unnecessary when margins are in enamel. Avoiding beveling in such situations contributes to preserve sound tooth structure and supports a more conservative restorative approach.

## Graphical abstract

**Figure:**
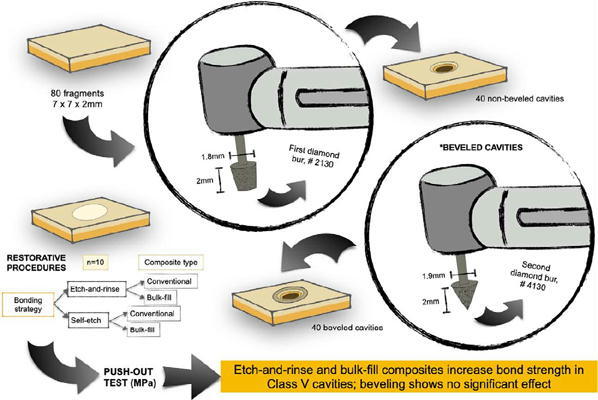

## Introduction

Cervical lesions are highly prevalent and may or may not be associated with dental caries. Due to their multifactorial nature, identifying the causes of non-carious cervical lesions (NCCLs) formation is complex, as they can result from excessive masticatory load, erosion, attrition, and/or traumatic tooth brushing.^1^, ^2^, ^3^ Restoring these cavities can help slow the progression of structural loss,^1^, ^2^ particularly in cases where periodontal surgery for root coverage is contraindicated. However, the location, tooth morphology, and cavity configuration appear to influence the longevity of adhesive restorations in cervical regions.^3^

Cavity configuration factor (C-factor) is one of the key elements affecting shrinkage stress on the cavity walls of Class V restorations.^4^ To minimize the C-factor impact, the incremental placement technique for resin composites has been proposed;^5^ however, this approach can be complex in small cavities with limited access, such as Class V lesions. An alternative restorative approach involves using bulk-fill composites, which exhibit lower polymerization shrinkage compared with conventional composites.^6^ Nonetheless, the effectiveness of these materials in cervical restorations remains uncertain.

What bonding strategy to use for adhesive restorations in cervical lesions is also a subject of debate.^7^, ^8^, ^9^ Currently, three main categories of bonding agents are available: etch-and-rinse systems, which require prior acid etching of enamel and dentin before adhesive application; self-etching systems, which promote chemical bonding to dentin through simultaneous demineralization and infiltration of the adhesive without requiring prior acid etching; and multi-mode or universal adhesives, which can be applied using either approach.^10^ The performance of these systems varies depending on several factors, including the degree of conversion of the adhesive agents, solvent volatilization, and the type of substrate being bonded.^4^, ^9^, ^10^

Historically, enamel beveling was introduced as a clinical procedure to improve the aesthetic integration of composite restorations.^2^, ^11^, ^12^ However, a previous study has shown that a bevel combined with a self-etching adhesive technique can reduce marginal microleakage in rectangular slot cavities restored on the buccal surface of molars,^11^ potentially minimizing discoloration over time. Since beveling can lead to unnecessary removal of sound enamel, assessing its influence on the bonding performance of Class V restorations with different adhesive strategies is essential to define its selective indication in modern restorative dentistry. Thus, this study assessed the influence of enamel bevel on the bonding performance of simulated cervical restorations made with conventional and bulk-fill resin composites, bonded with a universal adhesive using both the etch-and-rinse and self-etching strategies. Three hypotheses were tested: 1. Presence of enamel bevel would enhance bond strength; 2. Both bonding strategies (etch-and-rinse and self-etching) would provide similar bond strength to enamel/dentin cavities; 3. Bulk-fill composite would be a viable alternative to improve the bonding performance of the restoration.

## Methodology

### Specimen preparation

This study was approved by the Animal Ethics Committee (CEUA No. 2836220121). Bovine incisors were selected, cleaned, and subsequently immersed in a 0.5% thymol solution. A total of eighty blocks (7 mm × 7 mm) were sectioned from the buccal surface of the cervical region using diamond discs on a refrigerated cutting machine (Labcut 150, Extec, São Paulo, Brazil). The top (enamel) and bottom (dentin) surfaces of the blocks were flattened using 600-grit silicon carbide sandpaper mounted on a water-cooled polisher (APl-4, Arotec, Cotia, SP, Brazil) until achieving a standardized substrate thickness (1 mm enamel, 1 mm dentin). Blocks were then inspected for sample selection, and those showing cracks or structural defects were excluded.

Cervical lesions were simulated by preparing conical cavities (2.0 mm deep and 1.8 mm in diameter at their widest point) at the center of each block using highspeed conical diamond burs (#2130, KG Sorensen, Barueri, São Paulo, Brazil) under constant water cooling. This geometry ensured that the larger diameter remained at the enamel level. Burs were replaced after every five preparations to standardize cutting efficiency.

**Figure 1:**
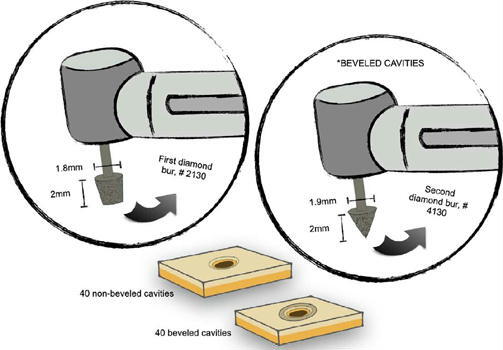
Schematic representation of specimen preparation and diamond bur characteristics.

A cavosurface bevel was then created exclusively in enamel using a cone-shaped diamond bur (#4130, Coraldent, São Paulo, Brazil). During beveling, only the upper portion of the bur, positioned at an approximate 45° angle relative to the enamel surface, contacted the cavity edges. This technique produced a bevel of approximately 1.0 mm in extension, consistent with clinical enamel beveling procedures performed under high-speed instrumentation. As the bevel was prepared freehand, this extension was later measured and used for accurate calculation of the bonded area. The burs used for beveling were also replaced after every five preparations. All blocks were stored in distilled water at room temperature (25 °C) for 24 hours before adhesive procedures. Figure 1 illustrates the specimen preparation process.

All cavity preparations were performed by a single calibrated operator to ensure methodological consistency. Operator calibration was confirmed via pilot procedures before the experimental phase. During cavity preparation, each enamel block was fixed in a custom metallic holder that maintained the long axis of the bur perpendicular to the enamel surface, ensuring accurate control of angulation and preventing the formation of oblique vectors that could affect the push-out bond strength results.

### Restorative procedures

The prepared blocks were randomly assigned to eight groups (n=10), based on bevel presence or absence, adhesive strategy (etch-and-rinse or self-etching), and resin composite type (conventional or bulk-fill). Sample size per group was determined by a sample size calculation procedure.

A universal dental adhesive (Ambar Universal APS, FGM, Joinville, Santa Catarina, Brazil) was applied according to the manufacturer’s instructions for both the self-etching and etch-and-rinse strategies. In the etch-and-rinse approach, both enamel and dentin were etched with 37% phosphoric acid (Condac 37%, FGM, Joinville, Santa Catarina, Brazil) for 15 seconds, followed by rinsing with an air/water spray for 15 seconds at 10 cm. Excess water was removed with absorbent paper to maintain appropriate moisture levels of the substrates. The adhesive was actively applied for 10 seconds in two layers, with excess material removed using a microbrush. A gentle air stream was applied for 10 seconds from 10 cm to volatilize the solvent. The light-curing unit (LCU) tip was positioned 1 mm from the specimen, and light activation was performed for 10 seconds (Valo, Ultradent, Itaici-Indaiatuba, São Paulo, Brazil) at an irradiance of 1,400 mW/cm^2^. In the self-etching mode, the adhesive was applied in the same manner to both surfaces, without prior acid etching.

Following the adhesive procedures, the cavities were restored with either a conventional nanohybrid composite resin (Opallis, FGM, Joinville, Santa Catarina, Brazil) in two 1-mm increments or a bulk-fill composite resin (Opus Bulk Fill APS, FGM, Joinville, Santa Catarina, Brazil) in a single 2-mm increment. The bulk-fill resin composite used presents conventional (non-flowable) viscosity, allowing comparable handling to the nanohybrid material. A Mylar strip (KDent, Quimidol, Joinville, Santa Catarina, Brazil) was placed over the restoration, and a 500 g weight was applied for 30 seconds to enhance resin accommodation. The LCU tip was then positioned in contact with the Mylar strip on the enamel side, and light activation was performed for 20 seconds.

The specimens were individually stored in Eppendorf tubes containing distilled water at 37°C for 24 hours. Subsequently, the restorations were finished and polished using abrasive discs (Sof-Lex Pop-On 4930 3/8, 3M, Sumaré, São Paulo, Brazil) in descending order of abrasiveness, ensuring uniform surface finishing and removal of any potential excess adhesive or composite material at the cavity margins.

### Push-out bond strength test

Push-out shear testing was conducted on a universal testing machine (2000RK, Kratos, São Paulo, Brazil). Block and restoration dimensions were confirmed using a digital caliper. Each specimen was fixed onto a base with a central hole (3 mm), ensuring that the larger cavity diameter was positioned opposite the load direction. The specimens were subjected to load until failure at a crosshead speed of 0.5 mm/min.

The bonded area for the push-out test was calculated according to the geometry produced by the conical preparation. For standard cavities, the adhesive interface corresponded to the lateral surface of a truncated cone and was calculated as follows:

A_cavity=π∗(r1+r2)∗g

in which n and r2 are the radii of the widest and narrowest portions of the cavity, and g is the slanted height derived from the measured cavity depth.

For the beveled groups, the cavosurface bevel created an additional conical surface. Since beveling was performed at approximately 45°, the bevel extension and its internal and external diameters were measured for each specimen. The bevel area was calculated as an additional truncated cone:

$$A\_bevel=\pi *\left(r1^{\prime }+r2^{\prime }\right)*g^{\prime }$$

Total bonded area was obtained by summing both components:

A_total=A_cavity+A_bevel

Push-out bond strength was calculated by dividing the failure load (N) by the total bonded area and expressed in MPa.

### Statistical analysis

Sample size was calculated on Bioestat 5.0, based on bond strength data from previous studies, considering a power of 0.8 and α = 0.05.

Collected data underwent exploratory data analysis to assess the assumptions for analysis of variance (ANOVA). The data followed a normal distribution (Shapiro-Wilk test, p=0.09) and exhibited homogeneity of variances (Levene’s test, p=0.432). Inferential statistical analysis was performed using a three-way ANOVA, considering the margin configuration, adhesive strategy, and composite type as the main factors. Tukey’s post-hoc test was used for multiple comparisons between means. All analyses were performed on JAMOVI statistical software, with a significance level set at 5%.

## Results

Table 1 presents the bond strength values (MPa) according to margin configuration, bonding strategy, and composite resin type.

**Table 1:** Bond strength values in MPa, mean (standard deviation) according to margin configuration, adhesive strategy, and composite resin type.

Margin configuration	Bonding strategy (MPa)	Composite resin	
		Bulk-fill	Conventional
Bevel	Etch&Rinse	13,2 (3,18)	9,85 (1,39)
	Self-etch	11,5 (3,27)	10,0 (1,44)
No bevel	Etch&Rinse	13,7 (1,83)	11,6 (2,02)
	Self-etch	12,3 (3,77)	9,95 (1,96)

**Figure 2:**
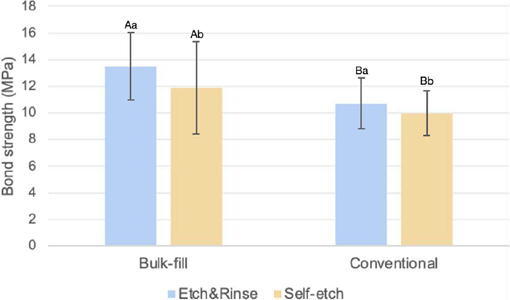
Bar chart showing bond strength values and comparisons between adhesive strategy and composite resin type (Data from margin configuration were pooled).

As no significant interaction was observed between the main factors (p>0.05), these were analyzed independently. Presence or absence of a margin bevel did not significantly affect bond strength (p=0.177); however, statistically significant differences were found for adhesive strategies (p=0.042) and resin composite types (p<0.001). Mean ± SD values revealed that specimens restored using the etch-and-rinse strategy showed superior bond strength compared with the self-etch approach. Additionally, the bulk-fill composite resin exhibited higher bond strength than the conventional composite resin in simulated cervical lesions (Figure 2).

Dissimilar lowercase letters mean significant differences between etch-and-rinse and self-etch groups and different uppercase letters mean significant differences between bulk-fill and conventional composite groups (three-way ANOVA/Tukey’s post-hoc test, 5%).

## Discussion

Deciding whether to use a marginal bevel and selecting the most effective adhesive strategy for cervical lesion restorations are common clinical concerns. As a primary objective, this study sought to provide clinicians with additional evidence regarding these issues. To the best of our knowledge, this is the first study to evaluate the bonding performance of universal adhesives and bulk-fill composites in simulated Class V cavities, simultaneously testing both enamel and dentin substrates.

Cervical lesions typically involve both enamel and dentin margins^2^, but all cavities in this study had exclusively enamel margins to ensure substrate homogeneity and consistent stress distribution during push-out bond strength testing. This was an intentional methodological choice as including dentin margins would have introduced additional variables like differences in elastic modulus, leading to non-uniform stress distribution and potentially confounding the results.

Although push-out testing is frequently used to assess the bond strength of endodontic cements in intraradicular canals, it can be efficiently adapted to evaluate the performance of restorative materials in cavities with a high C-factor.^13^ It simultaneously subjects surrounding cavity walls to load, enabling the evaluation of shear bond strength.^13^ Additionally, the method used here enabled evaluating restorations bonded to both enamel and dentin, replicating the clinical scenario of some cervical lesions which often involve both substrates. The push-out test was thus chosen instead of the commonly used microtensile test because it allows simultaneous loading of all surrounding cavity walls and maintains cavity integrity, which would not be feasible with microtensile testing.

Here, bevel did not improve the push-out bond strength of adhesive restorations. Consequently rejecting the first hypothesis. Patanjali, et al.^11^ (2019) reported that using bevel combined with a self-etching strategy reduced microleakage but did not eliminate it completely, suggesting that bevel could enhance bond strength when used with the etch-and-rinse system. Such discrepancies between our results and those of Patanjali, et al.^11^ (2019) may be attributed to methodological differences, including cavity geometry, bevel preparation (multilaminate vs. diamond burs), and restorative materials. Bevel protocols for cervical restorations remain poorly standardized in the literature, with variations in instrumentation and wear depth complicating direct comparisons.^1^, ^11^, ^12^, ^15^ Here we used a conical diamond bur to standardize the bevel and ensure consistent extension across all tested specimens; however, this finding should be interpreted with caution. In conservative dentistry, beveling is primarily indicated for aesthetic purposes and not as a strategy to enhance adhesion. For cervical lesions, which are often influenced by multifactorial conditions like masticatory load, erosion, attrition, and abrasive tooth brushing, beveling is not recommended to improve dentin bonding.

Previous studies have also reported that beveling does not significantly impact short-term retention rates or reduce marginal discoloration^1^ and does not prevent nanoleakage,^15^ despite its aesthetic advantages.^12^ Based on current and prior findings, beveling should be selectively used in cases requiring optimal aesthetics to conceal the resin-enamel interface. In non-aesthetic regions, beveling may be unnecessary and might be avoided to preserve healthy dental tissue.

The universal adhesive used here is classified as ultra-mild, with a pH of approximately 2.57. It contains 10-methacryloyloxydecyl dihydrogen phosphate (10-MDP), a functional monomer widely present in commercial self-etch and universal adhesives that forms stable calcium salts with hydroxyapatite.^16^ Presence of 10-MDP allows universal adhesives to achieve adequate bonding to dental hard tissues, even in self-etch mode.^7^, ^8^ However, the self-etch strategy used resulted in lower bond strength compared with the etch-and-rinse approach, thus rejecting the second hypothesis. Differences between the strategies may be attributed to the absence of selective enamel etching, a beneficial procedure when applying dental adhesives in self-etch mode.^17^, ^8^ Although the bond strength difference between etch-and-rinse and self-etching strategies was statistically significant, the absolute difference was relatively small, particularly when using conventional composites. However, a previous clinical study showed that using universal adhesives in the etch-and-rinse strategy resulted in higher clinical success after 36 months compared with the same adhesive applied by self-etching in non-carious cervical lesion restorations, even when selective enamel etching was performed, validating our findings regarding the adhesive strategy.^18^

Another factor that may enhance the adhesion of cervical restorations is the use of composites with low polymerization shrinkage.^19^ Bulk-fill composites offer several advantages, including simplified restorative procedures, reduced operative time, and minimized technical errors such as void incorporation, postoperative sensitivity, microleakage, and marginal discoloration.^19^, ^20^, ^21^

In this study, bulk-fill composites exhibited superior bond strength compared with conventional composites restored using an incremental technique, supporting the third hypothesis. Previous studies have shown that bulk-fill composites generate lower shrinkage stress in high C-factor cavities and present improved flexural properties compared with conventional composites.^4^, ^18^, ^22^ Reduced stress at the cavity walls and adhesive interface^9^ may explain the higher bond strength observed with bulk-fill composites.^23^ Similar findings have been reported in Class I restorations, where bulk-fill resins performed comparably or better than conventional composites.

Many Class V cavities are in aesthetically significant areas^24^ and the cervical region of teeth naturally exhibits greater opacity compared with the middle and incisal/occlusal thirds.^25^, ^26^ Due to their ability to polymerize in thick increments (4-5 mm), bulk-fill materials generally have higher translucency than conventional composites.^22^ Thus, using bulk-fill composites in aesthetic regions should be carefully considered, as they may not always achieve the ideal optical properties required for restoration.

Despite the inherent limitations of laboratory designs, such as simplified substrates and absence of intraoral aging, *in vitro* findings like those of this study provide valuable insights into adhesive performance under controlled conditions. Our data offer a foundational basis for future clinical investigations but should not be directly extrapolated to clinical practice. Further investigations incorporating different variables, failure mode analysis and long-term evaluations are necessary to complement the present findings, particularly regarding restoration longevity.

## Conclusion

Cervical lesions are common in clinical practice, and many Class V cavities can be effectively restored using composite resins. Using universal adhesives and bulk-fill resins in these cases represents a promising and simplified approach for clinicians, warranting further investigation. Our findings suggest that combining an etch-and-rinse strategy and a bulk-fill composite resin provides the highest bond strength in Class V restorations involving both enamel and dentin when a universal adhesive is used. Additionally, beveling of the enamel margin did not significantly enhance bond strength in this scenario. Thus, beveling should be reserved for aesthetic areas rather than routinely performed in adhesive Class V restorations.

## Data Availability

All data generated and analyzed during this study are included in this published article.
